# *CD99* polymorphisms significantly influence the probability to develop Ewing sarcoma in earlier age and patient disease progression

**DOI:** 10.18632/oncotarget.12862

**Published:** 2016-10-25

**Authors:** Marcella Martinelli, Alessandro Parra, Luca Scapoli, Paola De Sanctis, Valentina Chiadini, Claudia Hattinger, Piero Picci, Cinzia Zucchini, Katia Scotlandi

**Affiliations:** ^1^ Department of Experimental Diagnostics and Specialty Medicine (DIMES), University of Bologna, Bologna, Italy; ^2^ CRS Development of Biomolecular Therapies, Oncology Laboratory, Rizzoli Orthopaedic Institute, Bologna, Italy; ^3^ Experimental Oncology Laboratory, Rizzoli Orthopaedic Institute, Bologna, Italy

**Keywords:** CD99, Ewing sarcoma, polymorphisms, association analysis

## Abstract

Ewing sarcoma (EWS), the second most common primary bone tumor in pediatric age, is known for its paucity of recurrent somatic abnormalities. Apart from the chimeric oncoprotein that derives from the fusion of *EWS* and *FLI* genes, recent genome-wide association studies have identified susceptibility variants near the *EGR2* gene that regulate DNA binding of *EWS-FLI*. However, to induce transformation, *EWS-FLI* requires the presence of additional molecular events, including the expression of CD99, a cell surface molecule with critical relevance for the pathogenesis of EWS. High expression of CD99 is a common and distinctive feature of EWS cells, and it has largely been used for the differential diagnosis of the disease. The present study first links *CD99* germline genetic variants to the susceptibility of EWS development and its progression. In particular, a panel of 25 single nucleotide polymorphisms has been genotyped in a case-control study. The *CD9*9 rs311059 T variant was found to be significantly associated [*P* value = 0.0029; OR_het_ = 3.9 (95% CI 1.5-9.8) and OR_hom_ = 5.3 (95% CI 1.2-23.7)] with EWS onset in patients less than 14 years old, while the CD99 rs312257-T was observed to be associated [*P* value = 0.0265; OR_het_ = 3.5 (95% CI 1.3-9.9)] with a reduced risk of relapse. Besides confirming the importance of *CD99*, our findings indicate that polymorphic variations in this gene may affect either development or progression of EWS, leading to further understanding of this cancer and development of better diagnostics/prognostics for children and adolescents with this devastating disease.

## INTRODUCTION

Ewing's sarcoma (EWS) is a highly malignant musculoskeletal tumor that preferentially displays aggressive growth, with approximately 30% of patients harboring disseminated metastatic disease at the time of diagnosis, the most common sites being the lungs and/or bones [1]. EWS arises abruptly in pediatric age, with the peak of incidence in the second decade of life, and its natural history is still mostly unknown. The tumor is more common in Caucasians while it rarely appears in individuals of African or Asian heritage [2–6]. This observation together with reports indicating EWS in siblings or cousins [7, 8], suggests that genetic susceptibility factors may exist for this tumor, particularly among European population. However, due to the rarity of the disease, only sporadic information is available. Genome-wide scan for EWS susceptibility loci identified two common variants associated with higher susceptibility to EWS in French population [9]: a variant mapping in 1p36.22 and located proximal to the *TARDBP* gene (Tat activating regulatory DNA-binding protein, or *TDP-43*, transactive response DNA-binding protein) and a variant in 10q21, located in a 561kb LD block containing three described genes: *ADO* (encoding cysteamine dioxygenase), *ZNF365* (encoding zinc-finger protein 365) and *EGR2* (encoding early growth response protein 2). Mechanistic studies connecting expression of these gene variants to EWS susceptibility have clearly demonstrated a functional association between *EGR2* and the oncogenic protein of EWS [10], the chimera *EWS-FLI*. EWS is indeed characterized by a chromosomal translocation between the *EWS* (locus 22q12) and *ETS* transcription factor genes, especially *FLI1* (11q24) (occurring in 85% of cases), or *ERG* (21q22) (about 5-10% of cases) [11, 12]. *EWS-FLI* gene fusion leads to the formation of a chimeric protein that drives the malignant phenotype by affecting both gene transcription [13–15] and RNA splicing [16, 17] of specific downstream target genes. The crucial role of *EWS-FLI* in EWS pathogenesis has been confirmed also by the genomic landscape of EWS [18–20], which showed a very low somatic mutation rate in this neoplasia. Analysis of SNPs in the *EWS* gene revealed that the rs4820804 TT genotype could be associated with a higher propensity of *EWS* breakage, increasing the chances for the translocation to occur [21]. However, in recent years it has become clear that the *EWS-FLI* oncogenic activity is a necessary but insufficient condition. To induce transformation, *EWS-FLI* requires a permissive cellular background and the presence of additional molecular events, including disruption of p53 and CD99 expression [22, 23]. CD99 is a cell surface molecule of 32 KDa [24] encoded by the pseudoautosomal *MIC2* gene, which is involved in crucial biological processes like migration, cell death, transendothelial migration of leukocytes, differentiation of T cells and thymocytes and transport of surface molecules [25–32]. CD99 is constantly present at high levels in EWS cells [33, 34] and its detection is routinely used for differential diagnosis. The EWS-FLI oncogenic activity [35] is facilitated by CD99 [23] and consistently, EWS-FLI maintains high levels of CD99 expression [23, 36, 37] either directly, through binding of *CD99* promoter [23, 37, 38] or indirectly through miRNA regulation [39]. Abrogation of CD99 in EWS cells leads to terminal neural differentiation and severely reduces tumor growth and bone metastasis in mice [23], supporting a central role for CD99 in the pathogenesis of EWS.

In the present study, we examined the genetic influence of *CD99* polymorphisms on EWS susceptibility in a representative sample of the Italian population. We were specifically interested to identify, in a case-control study, any potential genetic markers associated with age of diagnosis and to establish whether *CD99* polymorphisms may influence the EWS disease progression. Analyses revealed for the first time evidence of two variant alleles in the *CD99* gene: one (rs311059-T) strongly associated with earlier onset of EWS, and the other one (rs312257-T) related to patient event-free survival (EFS).

## RESULTS

### Impact of the *CD99* SNPs on the risk to develop EWS

Genotyping was carried out on a cohort of 100 EWS patients (clinicopathological features are summarized in Table 1). The genotyping iPlex assay included 25 SNPs mapping on *CD99* gene. Nine SNPs (rs311036, rs311092, rs311095, rs312199, rs313089, rs5982836, rs6567640, rs2267799, and rs311088) were excluded from statistical analyses because genotyping success rate was lower than 90%; the remaining 16 SNPs were anyhow adequate to detect association between CD99 and the risk of EWS. Deviation from Hardy-Weinberg equilibrium was observed for the SNPs rs5939307 and rs5939113 in both case- and control-group (*P* value < 0.001), although no apparent technical genotyping issues could be highlighted. In both circumstances the observed heterozygote frequency was lower than expected. Data from these SNPs were anyway tested for association, taking into account that caution is needed in case of deviation from the null hypothesis.

**Table 1:** Clinicopathological features of 100 EWS patients

Characteristics	N.	%
**Gender**		
Female	37	37
Male	63	63
**Age** (3-45 years)		
≤ 14 years	35	35
> 14 years	65	65
**Location**		
Extremities	64	64
Pelvis	13	13
Central	23	23
**Metastasis at diagnosis**		
Yes	20	20
No	80	80
**Local treatment**		
Surgery	56	56
Surgery + RXT	22	22
RXT	21	21
Not Done	1	1
**Chemoprotocol**		
EWS-REN	17	17
ISG/SSG III	64	64
ISG/SSG IV	9	9
Other	10	10

No evidence of association with alleles or genotypes was observed when EWS cases and controls were compared (Supplementary Table S1). Similarly, analysis carried out on haplotypes did not mark any association between EWS and *CD99* SNP alleles (data not shown). However, when patients were sub-grouped by age (≤14 and >14 years old), a significantly higher frequency of the *CD99* rs311059-T variant was observed in those showing an earlier onset of the disease (*P* value = 0.0029) [OR_het_ = 3.9 (95% CI 1.5-9.8) and OR_hom_ = 5.3 (95% CI 1.2-23.7)] (Table 2). The association test was significant at the Bonferroni corrected threshold level for multiple testing (α < 0.0031). A similar trend was corroborated when the EWS patients with earlier onset of the disease were compared to the control group (Table 2).

**Table 2:** Association analysis between *CD99* polymorphisms and EWS age onset

SNP information		Genotype age ≤14			Genotype age >14			MAF		AA	Odds Ratio (95% CI)	
SNP ID	Alleles*	11	12	22	11	12	22	case ≤14	case >14	*P value*	OR_het_	OR_hom_
rs311057	G/A	15	16	0	41	19	1	0.26	0.17	0.1692	2.30 (0.95-5.61)	0.89 (0.03-23.09)
**rs311059**	C/T	9	21	5	38	23	4	0.44	0.24	**0.0029**	**3.86 (1.51-9.84)**	**5.28 (1.18-23.71)**
rs311060	C/G	24	11	0	39	24	2	0.16	0.22	0.3214	0.75 (0.31-1.79)	0.32 (0.02-7.00)
rs1136447	C/T	10	17	8	11	37	17	0.47	0.45	0.3130	0.51 (0.18-1.42)	0.52 (0.16-1.72)
rs6567640	C/G	12	18	5	16	40	9	0.40	0.45	0.5294	0.60 (0.24-1.52)	0.74 (0.20-2.79)
rs311074	A/G	11	12	12	23	30	12	0.46	0.42	0.1799	0.84 (0.31-2.23)	2.09 (0.71-6.13)

SNP information		Genotype age ≤14			Genotype control			MAF		AA	Odds Ratio (95% CI)	
SNP ID	Alleles	11	12	22	11	12	22	case ≤14	control	*P value*	OR_het_	OR_hom_
rs311057	G/A	15	16	0	95	33	9	0.26	0.19	0.2005	3.07 (1.37-6.89)	0.32 (0.02-5.86)
**rs311059**	C/T	9	21	5	81	49	17	0.44	0.28	**0.0093**	**3.86 (1.64-9.09)**	2.65 (0.79-8.89)
rs311060	C/G	24	11	0	83	59	5	0.16	0.23	0.1591	0.65 (0.29-1.42)	0.31 (0.02-5.80)
rs1136447	C/T	10	17	8	38	70	37	0.47	0.50	0.7059	0.92 (0.39-2.21)	0.82 (0.29-2.31)
rs6567640	C/G	12	18	5	40	81	26	0.40	0.45	0.4278	0.74 (0.33-1.69)	0.64 (0.20-2.03)
rs311074	A/G	11	12	12	46	78	23	0.46	0.42	0.1610	0.64 (0.26-1.58)	2.18 (0.84-5.69)

SNP information		Genotype age >14			Genotype control			MAF		AA	Odds Ratio (95% CI)	
SNP ID	Alleles	11	12	22	11	12	22	case >14	control	*P value*	OR_het_	OR_hom_
rs311057	G/A	41	19	1	95	33	9	0.17	0.19	0.7387	1.33 (0.68-2.62)	0.26 (0.03-2.10)
rs311059	C/T	38	23	4	81	49	17	0.24	0.28	0.3477	1.00 (0.53-1.87)	0.50 (0.16-1.60)
rs311060	C/G	39	24	2	83	59	5	0.22	0.23	0.6625	0.87 (0.47-1.59)	0.85 (0.16-4.58)
rs1136447	C/T	11	37	17	38	70	37	0.45	0.50	0.3471	1.83 (0.84-3.99)	1.59 (0.66-3.84)
rs6567640	C/G	16	40	9	40	81	26	0.45	0.45	0.9054	1.24 (0.62-2.47)	0.87 (0.33-2.25)
rs311074	A/G	23	30	12	46	78	23	0.42	0.42	0.9022	0.77 (0.40-1.48)	1.04 (0.44-2.46)

Abbreviations: SNP, single nucleotide polymorphism; MAF, minor allele frequency; AA, allelic association; OR_het_, odds ratio for heterozygote; OR_hom_, odds ratio for homozygote.

* Major allele is provided first.

In order to improve the coverage of exon 2 region, which contains the rs311059, four additional SNPs close to this polymorphism were selected and genotyped. No evidence of association was observed between these additional markers and the predisposition to EWS. However, haplotype analysis confirmed that the haplotype rs311059-T/rs311060-C showed the same level of association with the onset of EWS before 14 years of age [*P* = 0.0029; OR = 2.5 (95% CI 1.2-23.7)] that was previously found for the allele rs311059-T. Moreover, to verify that *CD99* polymorphisms specifically influence the EWS onset in pediatric age, the genotyping was extended to patients with OS (clinicopathological features are summarized in Supplementary Table S2), which also frequently affects children and adolescents. No correlation was found between *CD99* rs311059-T and the risk to develop OS (data not shown), further confirming that the association between this variant and higher probability of premature disease onset is a hallmark of EWS.

### Impact of the *CD99* SNPs on EWS disease progression and patient outcome

**Figure 1:**
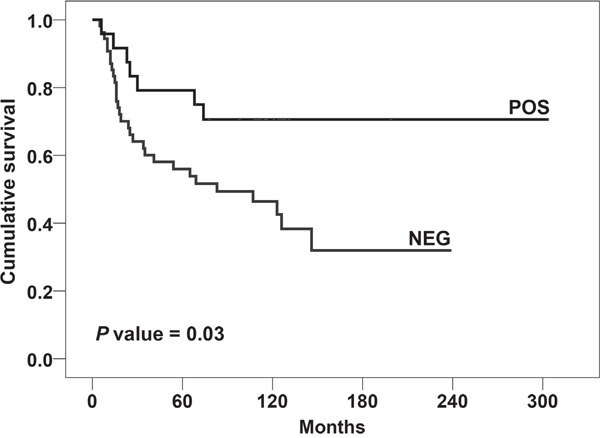
Prognostic impact of the presence of CD99 rs312257 T allele according to Kaplan–Meier curves and log-rank test EWS patients were classified for the presence (POS) or absence (NEG) of the variant. Event-free survival (EFS) was considered.

In the same cohort of EWS patients, we tested if *CD99* polymorphisms could influence disease progression. The value of *CD99* SNPs as prognostic biomarkers was calculated only in patients with localized EWS at diagnosis. Patients with metastatic disease at diagnosis were excluded from this analysis due to differences in treatment and the well-recognized negative impact of the presence of metastasis on patient outcome. The association analysis carried out on 78 patients with a minimum follow-up of 10 months (Table 3) showed that the variant rs312257-T is associated with a lower risk of EWS relapse [*P* = 0.0265; OR_het_ = 3.5 (95% CI 1.3-9.9)]. Although this link was not sufficiently strong to be confirmed after Bonferroni correction for multiple testing, the prognostic value of the *CD99* rs312257-T was proved to be associated with EFS of EWS patients when Kaplan-Meier survival curves were designed (Figure 1) (*P* = 0.03, log-rank test). Presence of rs312257 T variant allele as well as good response of tumors to neoadjuvant chemotherapy, which resulted significantly associated with clinical outcome in univariate analysis (Table 3), were confirmed as independent risk factors associated with good outcome by multivariate Cox's proportional hazards regression analysis (Table 4).

**Table 3:** Clinicopathological features of 78 localized EWS patients

Characteristics	N.	%	Association with prognosis EFS (*P* value)
**Gender**			
Female	31	39.7	0.760
Male	47	60.3	
**Age**			
≤ 14 years	27	34.6	0.160
> 14 years	51	65.4	
**Location**			
Extremities	53	67.9	0.637
Pelvis	18	23.1	
Central	7	9.0	
**Local treatment**			
Surgery	51	65.4	0.250
Surgery + RXT	17	21.8	
RXT	10	12.8	
**Chemoprotocol**			
EWS-REN	16	20.5	0.540
ISG/SSG III	58	74.4	
Other	4	5.1	
**Response to chemotherapy**			
Good	25	32.0	0.001
Poor	40	51.3	
ND	13	16.7	
**EFS (Status)**			
NED	39	50.0	
REL	39	50.0	

**Table 4:** Multivariate analysis using Cox's proportional hazards regression analysis

Variables associated with better prognosis	Adjusted risk-rate ratio	CI (95%)	*P* value
	EFS		
**Response to chemotherapy (GOOD)**	0.205	(0.078 – 0.539)	0.001
**Presence of T allele at rs312257 SNP**	0.657	(0.429 – 1.005)	0.053

Adjusted risk-rate (RR) ratio of relapse was estimated for the variables that resulted to be significantly associated with prognosis by univariated analysis.

## DISCUSSION

Although cancer is a complex disease resulting from the interplay of genetic, epigenetic, and environmental factors, the etiopathology of pediatric tumors supports the role of genetic and epigenetic alterations rather than environmental factors in the tumorigenesis. For EWS, race is one of the very few, well-recognized risk factor [40] and this supports the hypothesis of a genetic contribution to its etiology. However, familial cases of EWS are extraordinarily uncommon [7, 8, 41] and, therefore, any genetic predisposition would be expected to have low penetrance. Considering that approximately 95% of EWS patients harbor characteristic gene translocations involving the *EWS* gene, germline *EWS* SNPs have been studied by different groups without reporting genetic variations that significantly impact on susceptibility to develop EWS [21, 42]. Using a combination of high-throughput sequencing and integrative genomics, susceptibility variants near *EGR2* gene have been identified [9]. More recently, Grünewald *et al*. [10] has demonstrated that *EGR2* significantly influences EWS growth in mice and recognized an inherited variation on chromosome 10 that affects EWS susceptibility by facilitating the binding of the EWS-FLI oncoprotein to the *EGR2* locus. Besides *EWS-FLI*, the other major common determinant of EWS is CD99 [23, 43, 44], which contributes substantially to EWS-FLI transformation.

In this paper, we have evaluated *CD99* single nucleotide variations as potential risk factors for the development of EWS. We hypothesized that EWS occurring in pediatric age (i.e. ≤14 years) could be supported by a different genetic predisposition with respect to EWS occurring later in life. Indeed, we found that the *CD99* rs311059-T is significantly associated with an increased risk to develop the disease before 14 years of age. Rs311059 is a non-coding SNP and its functional relevance is unknown. As for the inherited variation on chromosome 10 near *EGR2* [10], *CD99* rs311059 variant allele may not affect the gene product sequence, but indirectly increasing susceptibility to EWS through epigenetic regulation of gene expression. Rs311059 maps inside the suppressor of zeste 12 (*SUZ12*) binding site, as shown on UCSC Genome Browser on Human Feb. 2009 (GRCh37/hg19) Assembly. *SUZ12*, a gene identified at the breakpoints of a recurrent chromosomal translocation reported in endometrial stromal sarcoma [45], encodes for a zinc finger protein belonging to the Polycomb Repressive Complex 2 (PRC2), which modulates chromatin structure by repressive mechanisms [46, 47]. PRC2 specifically stimulates H3K27 trimethylation, which is associated with transcriptional repression, and can recruit DNMTs [48], thus acting as a platform for aberrant *de novo* promoter methylation. We speculate that the presence of the rs311059 T allele creates a mismatch in SUZ12 binding site, which might facilitate the expression of *CD99*, enabling the permissive condition for EWS-FLI transformation. This could explain why rs311059-T appears to be a susceptibility factor in the EWS onset in pediatric age. The significant level of rs311059-T presence in young patients was found to be a distinctive feature of this disease. In fact, in patients affected by OS, which shares with EWS both the skeletal localization and the peak onset in childhood and adolescence age, the genotyping test did not provide any evidence of association.

In EWS, CD99 is expressed at high levels in virtually all samples [34, 49] but no correlation between CD99 expression and patient outcome has been highlighted so far. In this paper, we first identified the *CD99* rs312257 T genetic variant as significantly associated with EFS. The presence of the T allele confers superior survival to patients with localized EWS. Although mechanistic studies are needed to explain this observation, our findings support the hypothesis that variations in *CD99* gene may significantly affect the progression of EWS. Considering the rarity of the tumor, we offer this evidence to the scientific community for more extensive validation studies.

## MATERIALS AND METHODS

### Clinical samples

A cohort of 100 unrelated Italian patients with confirmed diagnosis of localized (80 cases) or disseminated (20 cases) EWS treated at the Rizzoli Orthopaedic Institute (Bologna, Italy) was considered. All cases included showed a specific EWS-ETS fusion, EWS patients underwent local treatments (surgery; surgery plus radiotherapy; radiotherapy only, when the surgeon considered the lesion inoperable or due to patient refusal) and neo-adjuvant chemotherapy according to protocols that were previously reported in detail [50, 51]. Clinicopathological data are shown in Table 1.

Patients with localized EWS were followed-up and clinical information updated (median follow-up: 76 months, range: 5-304 months). EFS was calculated from the date of initial diagnosis to clinical endpoint, which is considered as the time of occurrence of adverse events (defined as recurrence or metastases at any site for EFS). Histological response to chemotherapy was evaluated according to the method proposed by Picci et al. [52].

The control group consisted of 147 unrelated healthy volunteers with matching sex, ethnic origin and from the same geographical area (Italy). In addition, a cohort of 121 osteosarcoma (OS) samples was also enrolled for this study as a further comparison group. OS was chosen because it shares with EWS the same site of origin (bone) and a similar peak of incidence in juvenile age, despite being a very different type of tumor either from biological and genetic point of view [53]. Clinicopathological data of OS samples are shown in Supplementary Table S2. The ethical committee of the Rizzoli Institute approved the studies and informed consent was obtained.

### Single nucleotide polymorphism genotyping

DNA was extracted from peripheral blood leukocytes using standard DNAzol procedure (Thermo Fisher Scientific, Foster City, CA, USA). DNA quality and concentration were evaluated by Nanodrop (Thermo Fisher Scientific). Aliquots of 20 μl at the concentration of 12 ng/μl for each sample were employed for genotyping by the Sequenom MALDI-TOF mass spectrometer MassArray system (as a service at Applied Biomedical Research Center, S. Orsola-Malpighi Polyclinic, Bologna, Italy). To perform genotyping, the genotypic data of Caucasian and Italian population, collected by the International HapMap Consortium (CEU+TSI dataset), were evaluated by the HaploTagger software to explore the haplotype complexity of CD99 locus. We imposed that tag SNPs had to capture at least 80% of the CD99 alleles having a minor allele frequency > 0.1. We selected, therefore, 25 tag SNPs that best represent the genomic structure of CD99 with the minimal redundancy level [54]. Supplementary Figure S1 shows the position of each of the 25 SNPs along the CD99 locus and haploblock structures [54]. Assay design and analysis of allele peaks were performed using specific Sequenom software (Sequenom, San Diego, California, USA). Primers were synthesized at Metabion (Martinsried, Germany). In order to improve the coverage of the gene, the genotyping of 4 additional SNPs selected by Haplotagger was carried out by using a high resolution melting curve (HRM) analysis approach. Ten ng of DNA from each sample was amplified by using the MeltDoctor™ HRM Master Mix and the Applied Biosystems ViiA™ 7 Real-Time PCR System (Thermo Fisher Scientific), according to supplier's suggestions. The sequence of control samples was assessed by Sanger Sequencing and used as reference melt curve profile for the different genotypes.

### Statistical analysis

The deviations from Hardy-Weinberg equilibrium for genotype distributions, in both patient and control groups, were examined using Pearson's χ^2^ test.

Analysis for genotypic and haplotypic associations were performed using UNPHASED program (Version 3.1.7) which employs an allelic likelihood ratio test [55]. Odd ratios were calculated in order to estimate the level of association of the rare allele carriers, i.e. heterozygotes *vs* non-carriers, as well as homozygotes *vs* non carriers. The results were adjusted for multiple testing with the Bonferroni correction according to the number of tag SNPs analyzed. Bonferroni correction is considered an overly stringent adjustment when tests are not independent, such as in case of linkage disequilibrium between SNPs.

Patients were then sub-grouped by age at diagnosis into 2 groups (≤14 and >14 years old) and association analysis was performed. Comparisons as follows: 1) ≤14 *vs* >14 years old; 2) ≤14 years old *vs* control group; 3) >14 years old *vs* control group. The association was also tested for 4 additional SNPs (rs1136447, rs311060, rs6567640, and rs311074) selected by Haplotagger, covering the exon 2 and the boundaries. These four SNPs together with rs311057 and rs311059, mapping near to the 5′ exon 2 boundary were considered for haplotype analysis (until six marker combinations).

To verify association between *CD99* SNPs and patient prognosis, a subgroup of 78 patients with localized EWS having a minimum follow-up of 10 months was considered. For this subgroup, Kaplan-Meier and log-rank tests were used to draw and evaluate the significance of survival curves in EWS patients in relation to *CD99* SNP alleles.

## SUPPLEMENTARY MATERIALS TABLES AND FIGURE



## References

[1] Burchill SA (2003). Ewing's sarcoma: diagnostic, prognostic, and therapeutic implications of molecular abnormalities. Journal of clinical pathology.

[2] Linden G, Dunn JE (1970). Ewing's sarcoma in Negroes. Lancet.

[3] Jensen RD, Drake RM (1970). Rarity of Ewing's tumour in Negroes. Lancet.

[4] Li FP, Tu JT, Liu FS, Shiang EL (1980). Rarity of Ewing's sarcoma in China. Lancet.

[5] Jawad MU, Cheung MC, Min ES, Schneiderbauer MM, Koniaris LG, Scully SP (2009). Ewing sarcoma demonstrates racial disparities in incidence-related and sex-related differences in outcome: an analysis of 1631 cases from the SEER database, 1973-2005. Cancer.

[6] Worch J, Cyrus J, Goldsby R, Matthay KK, Neuhaus J, DuBois SG (2011). Racial differences in the incidence of mesenchymal tumors associated with EWSR1 translocation. Cancer epidemiology, biomarkers & prevention.

[7] Hutter RV, Francis KC, Foote FW (1964). Ewing's Sarcoma in Siblings: Report of the Second Known Occurrence. American journal of surgery.

[8] Joyce MJ, Harmon DC, Mankin HJ, Suit HD, Schiller AL, Truman JT (1984). Ewing's sarcoma in female siblings. A clinical report and review of the literature. Cancer.

[9] Postel-Vinay S, Veron AS, Tirode F, Pierron G, Reynaud S, Kovar H, Oberlin O, Lapouble E, Ballet S, Lucchesi C, Kontny U, Gonzalez-Neira A, Picci P (2012). Common variants near TARDBP and EGR2 are associated with susceptibility to Ewing sarcoma. Nature genetics.

[10] Grunewald TG, Bernard V, Gilardi-Hebenstreit P, Raynal V, Surdez D, Aynaud MM, Mirabeau O, Cidre-Aranaz F, Tirode F, Zaidi S, Perot G, Jonker AH, Lucchesi C (2015). Chimeric EWSR1-FLI1 regulates the Ewing sarcoma susceptibility gene EGR2 via a GGAA microsatellite. Nature genetics.

[11] Delattre O, Zucman J, Plougastel B, Desmaze C, Melot T, Peter M, Kovar H, Joubert I, de Jong P, Rouleau G (1992). Gene fusion with an ETS DNA-binding domain caused by chromosome translocation in human tumours. Nature.

[12] Zucman-Rossi J, Batzer MA, Stoneking M, Delattre O, Thomas G (1997). Interethnic polymorphism of EWS intron 6: genome plasticity mediated by Alu retroposition and recombination. Human genetics.

[13] Jedlicka P (2010). Ewing Sarcoma, an enigmatic malignancy of likely progenitor cell origin, driven by transcription factor oncogenic fusions. International journal of clinical and experimental pathology.

[14] Erkizan HV, Uversky VN, Toretsky JA (2010). Oncogenic partnerships: EWS-FLI1 protein interactions initiate key pathways of Ewing's sarcoma. Clin Cancer Res.

[15] Toomey EC, Schiffman JD, Lessnick SL (2010). Recent advances in the molecular pathogenesis of Ewing's sarcoma. Oncogene.

[16] Chansky HA, Hu M, Hickstein DD, Yang L (2001). Oncogenic TLS/ERG and EWS/Fli-1 fusion proteins inhibit RNA splicing mediated by YB-1 protein. Cancer research.

[17] Sanchez G, Delattre O, Auboeuf D, Dutertre M (2008). Coupled alteration of transcription and splicing by a single oncogene: boosting the effect on cyclin D1 activity. Cell cycle.

[18] Tirode F, Surdez D, Ma X, Parker M, Le Deley MC, Bahrami A, Zhang Z, Lapouble E, Grossetete-Lalami S, Rusch M, Reynaud S, Rio-Frio T, Hedlund E (2014). Genomic landscape of Ewing sarcoma defines an aggressive subtype with co-association of STAG2 and TP53 mutations. Cancer discovery.

[19] Crompton BD, Stewart C, Taylor-Weiner A, Alexe G, Kurek KC, Calicchio ML, Kiezun A, Carter SL, Shukla SA, Mehta SS, Thorner AR, de Torres C, Lavarino C (2014). The genomic landscape of pediatric Ewing sarcoma. Cancer discovery.

[20] Brohl AS, Solomon DA, Chang W, Wang J, Song Y, Sindiri S, Patidar R, Hurd L, Chen L, Shern JF, Liao H, Wen X, Gerard J (2014). The genomic landscape of the Ewing Sarcoma family of tumors reveals recurrent STAG2 mutation. PLoS genetics.

[21] Silva DS, Sawitzki FR, De Toni EC, Graebin P, Picanco JB, Abujamra AL, de Farias CB, Roesler R, Brunetto AL, Alho CS (2012). Ewing's sarcoma: analysis of single nucleotide polymorphism in the EWS gene. Gene.

[22] Lessnick SL, Dacwag CS, Golub TR (2002). The Ewing's sarcoma oncoprotein EWS/FLI induces a p53-dependent growth arrest in primary human fibroblasts. Cancer cell.

[23] Rocchi A, Manara MC, Sciandra M, Zambelli D, Nardi F, Nicoletti G, Garofalo C, Meschini S, Astolfi A, Colombo MP, Lessnick SL, Picci P, Scotlandi K (2010). CD99 inhibits neural differentiation of human Ewing sarcoma cells and thereby contributes to oncogenesis. The Journal of clinical investigation.

[24] Gelin C, Aubrit F, Phalipon A, Raynal B, Cole S, Kaczorek M, Bernard A (1989). The E2 antigen, a 32 kd glycoprotein involved in T-cell adhesion processes, is the MIC2 gene product. The EMBO journal.

[25] Bernard G, Breittmayer JP, de Matteis M, Trampont P, Hofman P, Senik A, Bernard A (1997). Apoptosis of immature thymocytes mediated by E2/CD99. Journal of immunology.

[26] Bernard G, Raimondi V, Alberti I, Pourtein M, Widjenes J, Ticchioni M, Bernard A (2000). CD99 (E2) up-regulates alpha4beta1-dependent T cell adhesion to inflamed vascular endothelium under flow conditions. European journal of immunology.

[27] Sohn HW, Shin YK, Lee IS, Bae YM, Suh YH, Kim MK, Kim TJ, Jung KC, Park WS, Park CS, Chung DH, Ahn K, Kim IS (2001). CD99 regulates the transport of MHC class I molecules from the Golgi complex to the cell surface. Journal of immunology.

[28] Pettersen RD, Bernard G, Olafsen MK, Pourtein M, Lie SO (2001). CD99 signals caspase-independent T cell death. Journal of immunology.

[29] Schenkel AR, Mamdouh Z, Chen X, Liebman RM, Muller WA (2002). CD99 plays a major role in the migration of monocytes through endothelial junctions. Nature immunology.

[30] Imbert AM, Belaaloui G, Bardin F, Tonnelle C, Lopez M, Chabannon C (2006). CD99 expressed on human mobilized peripheral blood CD34+ cells is involved in transendothelial migration. Blood.

[31] Lou O, Alcaide P, Luscinskas FW, Muller WA (2007). CD99 is a key mediator of the transendothelial migration of neutrophils. Journal of immunology.

[32] Bremond A, Meynet O, Mahiddine K, Coito S, Tichet M, Scotlandi K, Breittmayer JP, Gounon P, Gleeson PA, Bernard A, Bernard G (2009). Regulation of HLA class I surface expression requires CD99 and p230/golgin-245 interaction. Blood.

[33] Llombart-Bosch A, Machado I, Navarro S, Bertoni F, Bacchini P, Alberghini M, Karzeladze A, Savelov N, Petrov S, Alvarado-Cabrero I, Mihaila D, Terrier P, Lopez-Guerrero JA (2009). Histological heterogeneity of Ewing's sarcoma/PNET: an immunohistochemical analysis of 415 genetically confirmed cases with clinical support. Virchows Archiv.

[34] Kavalar R, Pohar Marinsek Z, Jereb B, Cagran B, Golouh R (2009). Prognostic value of immunohistochemistry in the Ewing's sarcoma family of tumors. Medical science monitor.

[35] Lessnick SL, Ladanyi M (2012). Molecular pathogenesis of Ewing sarcoma: new therapeutic and transcriptional targets. Annual review of pathology.

[36] Miyagawa Y, Okita H, Nakaijima H, Horiuchi Y, Sato B, Taguchi T, Toyoda M, Katagiri YU, Fujimoto J, Hata J, Umezawa A, Kiyokawa N (2008). Inducible expression of chimeric EWS/ETS proteins confers Ewing's family tumor-like phenotypes to human mesenchymal progenitor cells. Molecular and cellular biology.

[37] Hu-Lieskovan S, Zhang J, Wu L, Shimada H, Schofield DE, Triche TJ (2005). EWS-FLI1 fusion protein up-regulates critical genes in neural crest development and is responsible for the observed phenotype of Ewing's family of tumors. Cancer research.

[38] Amaral AT, Manara MC, Berghuis D, Ordonez JL, Biscuola M, Lopez-Garcia MA, Osuna D, Lucarelli E, Alviano F, Lankester A, Scotlandi K, de Alava E (2014). Characterization of human mesenchymal stem cells from ewing sarcoma patients. Pathogenetic implications. PloS one.

[39] Franzetti GA, Laud-Duval K, Bellanger D, Stern MH, Sastre-Garau X, Delattre O (2013). MiR-30a-5p connects EWS-FLI1 and CD99, two major therapeutic targets in Ewing tumor. Oncogene.

[40] Gurney JG, Swensen AR, Bulterys M, Ries LAG, Smith MA, Gurney JG, Linet M, Tamra T, Young JL, Bunin GR (1999). Malignant Bone Tumors. Cancer Incidence and Survival among Children and Adolescents: United States SEER Program 1975-1995.

[41] Zamora P, Garcia de Paredes ML, Gonzalez Baron M, Diaz MA, Escobar Y, Ordonez A, Lopez Barea F, Gonzalez JM (1986). Ewing's tumor in brothers. An unusual observation. American journal of clinical oncology.

[42] DuBois SG, Goldsby R, Segal M, Woo J, Copren K, Kane JP, Pullinger CR, Matthay KK, Witte J, Lessnick SL, Robison LL, Bhatia S, Strong LC (2012). Evaluation of polymorphisms in EWSR1 and risk of Ewing sarcoma: a report from the Childhood Cancer Survivor Study. Pediatric blood & cancer.

[43] Scotlandi K, Baldini N, Cerisano V, Manara MC, Benini S, Serra M, Lollini PL, Nanni P, Nicoletti G, Bernard G, Bernard A, Picci P (2000). CD99 engagement: an effective therapeutic strategy for Ewing tumors. Cancer research.

[44] Guerzoni C, Fiori V, Terracciano M, Manara MC, Moricoli D, Pasello M, Sciandra M, Nicoletti G, Gellini M, Dominici S, Chiodoni C, Fornasari PM, Lollini PL (2015). CD99 triggering in Ewing sarcoma delivers a lethal signal through p53 pathway reactivation and cooperates with doxorubicin. Clin Cancer Res.

[45] Koontz JI, Soreng AL, Nucci M, Kuo FC, Pauwels P, van Den Berghe H, Dal Cin P, Fletcher JA, Sklar J (2001). Frequent fusion of the JAZF1 and JJAZ1 genes in endometrial stromal tumors. Proceedings of the National Academy of Sciences of the United States of America.

[46] Schuettengruber B, Cavalli G (2009). Recruitment of polycomb group complexes and their role in the dynamic regulation of cell fate choice. Development.

[47] Simon JA, Kingston RE (2009). Mechanisms of polycomb gene silencing: knowns and unknowns. Nature reviews Molecular cell biology.

[48] Vire E, Brenner C, Deplus R, Blanchon L, Fraga M, Didelot C, Morey L, Van Eynde A, Bernard D, Vanderwinden JM, Bollen M, Esteller M, Di Croce L (2006). The Polycomb group protein EZH2 directly controls DNA methylation. Nature.

[49] Kovar H, Dworzak M, Strehl S, Schnell E, Ambros IM, Ambros PF, Gadner H (1990). Overexpression of the pseudoautosomal gene MIC2 in Ewing's sarcoma and peripheral primitive neuroectodermal tumor. Oncogene.

[50] Ferrari S, Sundby Hall K, Luksch R, Tienghi A, Wiebe T, Fagioli F, Alvegard TA, Brach Del Prever A, Tamburini A, Alberghini M, Gandola L, Mercuri M, Capanna R (2011). Nonmetastatic Ewing family tumors: high-dose chemotherapy with stem cell rescue in poor responder patients. Results of the Italian Sarcoma Group/Scandinavian Sarcoma Group III protocol. Annals of oncology.

[51] Rosito P, Mancini AF, Rondelli R, Abate ME, Pession A, Bedei L, Bacci G, Picci P, Mercuri M, Ruggieri P, Frezza G, Campanacci M, Paolucci G (1999). Italian Cooperative Study for the treatment of children and young adults with localized Ewing sarcoma of bone: a preliminary report of 6 years of experience. Cancer.

[52] Picci P, Bohling T, Bacci G, Ferrari S, Sangiorgi L, Mercuri M, Ruggieri P, Manfrini M, Ferraro A, Casadei R, Benassi MS, Mancini AF, Rosito P (1997). Chemotherapy-induced tumor necrosis as a prognostic factor in localized Ewing's sarcoma of the extremities. Journal of clinical oncology.

[53] Helman LJ, Meltzer P (2003). Mechanisms of sarcoma development. Nature reviews Cancer.

[54] de Bakker PI, Yelensky R, Pe'er I, Gabriel SB, Daly MJ, Altshuler D (2005). Efficiency and power in genetic association studies. Nature genetics.

[55] Dudbridge F (2008). Likelihood-based association analysis for nuclear families and unrelated subjects with missing genotype data. Human heredity.

