# Supplementary material for: CD99 polymorphisms significantly influence the probability to develop Ewing sarcoma in earlier age and patient disease progression
Source: Oncotarget. 2016 Oct 25;7(47):77958–67. doi: 10.18632/oncotarget.12862 (PMC5363635; doi:10.18632/oncotarget.12862)
Supplement: Supplementary file 1 [file oncotarget-07-77958-s001.pdf]

CD99 polymorphisms significantly influence the probability to develop Ewing sarcoma in earlier age and patient disease progression

SUPPLEMENTARY TABLES AND FIGURE

Supplementary Table S1: Association analysis between *CD99* polymorphisms and EWS

SNP information		Genotype count case			Genotype count control			MAF		AA	Odds Ratio (95% CI)	
SNP ID	Alleles*	11	12	22	11	12	22	case	control	*P* value	OR_{het}	OR_{hom}
rs2109560	C/T	46	41	8	62	70	12	0.30	0.33	0.54	0.79 (0.46-1.36)	0.90 (0.34-2.38)
rs167453	G/T	37	53	10	64	63	20	0.36	0.35	0.74	1.46 (0.84-2.51)	0.87 (0.37-2.04)
rs5982572	A/G	26	55	18	49	67	30	0.46	0.43	0.59	1.55 (0.85-2.80)	1.13 (0.53-2.40)
rs311057	A/G	56	35	1	95	33	9	0.20	0.19	0.69	1.80 (1.01-3.21)	0.19 (0.02-1.53)
rs311059	C/T	47	44	9	81	49	17	0.31	0.28	0.51	1.55 (0.90-2.66)	0.91 (0.38-2.21)
rs7051896	A/T	38	43	14	52	67	24	0.37	0.40	0.53	0.88 (0.50-1.55)	0.80 (0.37-1.74)
rs311077	C/G	55	35	10	78	57	12	0.28	0.28	0.99	0.87 (0.51-1.51)	1.18 (0.48-2.93)
rs5939307	C/T	58	30	12	84	45	17	0.27	0.27	0.99	0.97 (0.55-1.71)	1.02 (0.45-2.30)
rs5939113	A/G	71	17	5	98	23	8	0.15	0.15	0.86	1.02 (0.51-2.05)	0.86 (0.27-2.75)
rs311083	C/G	34	56	10	63	63	21	0.38	0.36	0.60	1.65 (0.95-2.86)	0.88 (0.37-2.09)
rs178127	G/T	47	39	10	63	58	17	0.31	0.33	0.55	0.90 (0.52-1.57)	0.79 (0.33-1.88)
rs312258	A/C	63	33	4	79	55	13	0.21	0.28	0.07	0.75 (0.44-1.30)	0.39 (0.12-1.24)
rs312257	C/T	73	27	0	112	31	4	0.14	0.13	0.94	1.34 (0.74-2.42)	0.17 (0.01-3.21)
rs311097	C/G	43	47	10	55	68	20	0.34	0.38	0.33	0.88 (0.51-1.53)	0.64 (0.27-1.51)
rs311100	A/G	54	39	7	79	54	13	0.27	0.27	0.83	1.06 (0.62-1.81)	0.79 (0.30-2.10)
rs5939312	A/G	74	25	1	111	35	1	0.14	0.13	0.77	1.07 (0.59-1.94)	1.50 (0.09-24.36)

Abbreviations: SNP, single nucleotide polymorphism; MAF, minor allele frequency; AA, allelic association; OR_{het}, odds ratio for heterozygote; OR_{hom}, odds ratio for homozygote.
*Major allele is provided first.

Supplementary Table S2: Clinicopathological features of 121 patients with primary osteosarcoma

Characteristics	N.	%
**Gender**		
Female	44	36
Male	77	64
**Age**		
≤ 14 years	28	23
> 14 years	93	77
**Location**		
Extremity	111	92
Other	10	8
**Histological subtypes**		
Osteoblastic	88	73
Fibroblastic	14	11
Others	19	16
**Surgery**		
Resection	103	85
Amputation	11	9
Not done	7	6

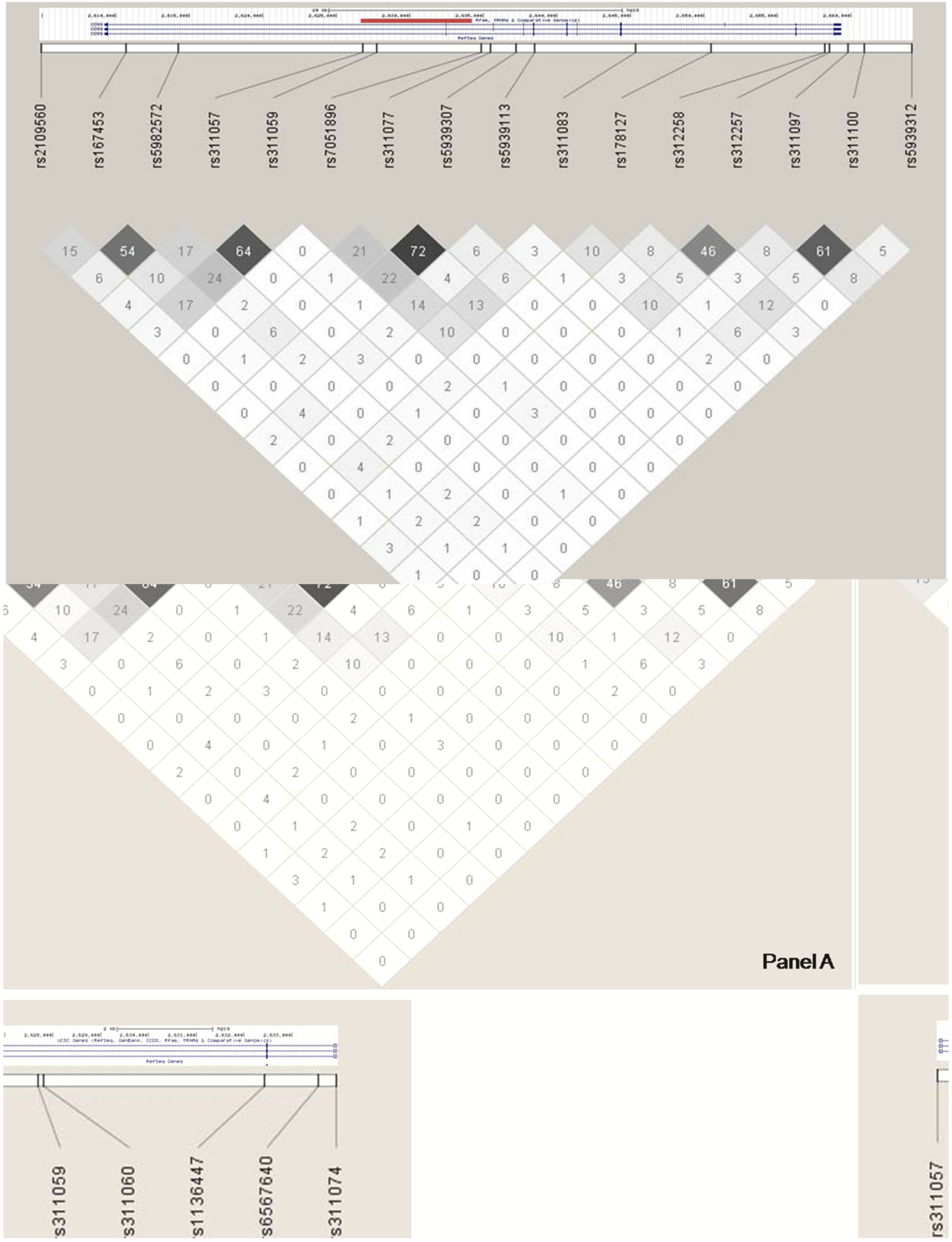

Supplementary Figure S1: Linkage disequilibrium plots of the investigated area; numbers in squares indicate r² index. **Panel A:** map of 16 CD99 SNPs used for association analysis with EWS. Red line represents the region around exon 2 investigated with additional SNPs (Panel B). **Panel B:** plot of the region nearby exon 2, investigated with 4 additional SNPs by HRM. Numbers in squares indicate r² index.
